# Successful management of a congenital cervical teratoma exhibiting rapid growth and pharyngeal adhesion

**DOI:** 10.1093/jscr/rjaf887

**Published:** 2025-12-04

**Authors:** Alysala M Malik, Yeu Sanz Wu, Tom Ben Dov, June K Wu, Salvatore M Caruana, Eli Grunstein, Russell S Miller, Vincent P Duron

**Affiliations:** Columbia University Vagelos College of Physicians and Surgeons, 630 W 168th St, NYC, NY 10032, United States; Division of Pediatric Surgery, New York Presbyterian Morgan Stanley Children’s Hospital of New York, 3959 Broadway, NYC, NY 10032, United States; Division of Pediatric Otolaryngology, New York Presbyterian Morgan Stanley Children’s Hospital of New York, 3959 Broadway, NYC, NY 10032, United States; Division of Plastic Surgery, New York Presbyterian Morgan Stanley Children’s Hospital of New York, 3959 Broadway, New York City, NY 10032, United States; Division of Otolaryngology, Columbia University Medical Center, 622 W 168th St, New York City, NY 10032, United States; Division of Pediatric Otolaryngology, New York Presbyterian Morgan Stanley Children’s Hospital of New York, 3959 Broadway, NYC, NY 10032, United States; Division of Maternal Fetal Medicine, Columbia University Medical Center, 622 W 168th St, New York City, NY 10032, United States; Division of Pediatric Surgery, New York Presbyterian Morgan Stanley Children’s Hospital of New York, 3959 Broadway, NYC, NY 10032, United States

**Keywords:** congenital cervical teratoma, neonate, laryngeal invasion, ex-utero intrapartum treatment (EXIT) pediatric surgery, otolaryngology

## Abstract

Congenital cervical teratomas are rare anomalies that are associated with a high rate of neonatal mortality if left untreated. Perinatal airway stabilization and prompt surgical management are imperative for survival and positive long-term outcomes. Poor prenatal prognostic factors include rapid teratoma growth, airway compression, polyhydramnios, and abnormal fetal breathing. We present the case of a neonate with an exceptionally large cervical teratoma (914 ml) with laryngeal compression and dense pharyngeal adhesions. Prenatally, the teratoma exhibited a period of rapid growth, abnormal fetal breathing, and recurrent severe polyhydramnios. The fetus required an ex-utero intrapartum treatment procedure for airway stabilization. The neonate underwent a successful surgical resection at eight days of life with a multidisciplinary team that included pediatric surgery, otolaryngology, and plastic surgery. At 15 months of life, the infant is doing well at home with a gastrostomy tube and tracheostomy collar.

## Introduction

Cervical teratomas are rare congenital anomalies with an incidence of 1 in 20 000 to 40 000 live births [1]. If untreated, they are associated with a > 80% mortality rate due to airway complications at birth. Significant maternal health risks are also seen due to challenges associated with fetal delivery [1–4]. Prenatal factors associated with the need for emergent airway requirements at birth include airway compression, rapid teratoma growth rates, abnormal fetal breathing, and polyhydramnios [1, 5–7]. Improvement in prenatal detection and management of cervical teratomas has resulted in drastically reduced neonatal mortality [8]. We present the case of a fetus with a large cervical teratoma causing laryngeal obstruction that was successfully delivered by ex-utero intrapartum treatment (EXIT) procedure and managed by a multidisciplinary team involving maternal–fetal medicine, pediatric surgery, otolaryngology, plastic surgery, radiology, and anesthesiology.

## Case report

**Figure 1:**
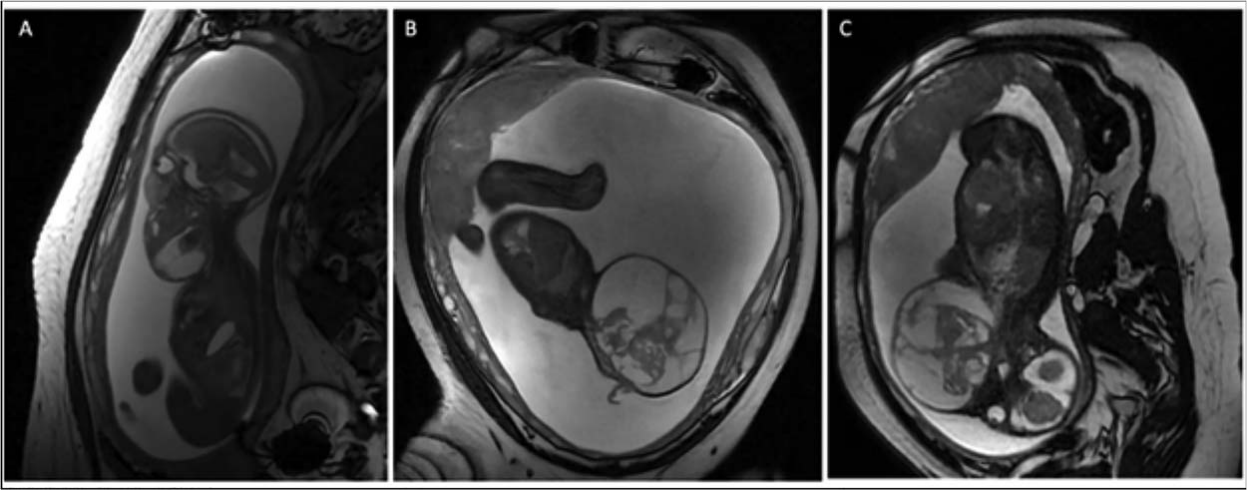
(A) Sagittal T2 weighted fetal MRI at 21 weeks gestation with fetal mass volume of 33 ml (3.7 × 4.7 × 3.6 cm). (B) Sagittal T2 weighted fetal MRI at 29 weeks gestation with fetal mass volume of 411 ml (10.3 × 9.3 × 8.2 cm). (C) Coronal T2 weighted fetal MRI at 29 weeks demonstrating fetal neck hyperextension due to mass effect.

A 28-year-old G2P1001 female presented for a routine 20-week gestation ultrasound, which demonstrated a left-sided fetal neck mass suspicious for lymphatic malformation versus cervical teratoma. Fetal single nucleotide polymorphism (SNP) oligonucleotide microarray and chromosomal fluorescence in-situ hybridization analyses showed no abnormalities. A fetal magnetic resonance imaging (MRI) at 21-weeks demonstrated a complex, multi-cystic and solid mass emanating from the left cervical region, crossing the midline anteriorly, and causing laryngeal compression (Fig. 1). No fetal breathing was seen on colour Doppler. A subsequent fetal MRI at 29-weeks showed a >10-fold increase in the size of the mass (33 to 411 ml) and complete airway occlusion (Fig. 1). Interval surveillance ultrasounds showed continued enlargement to an estimated volume of 914 ml at 32-weeks and persistent polyhydramnios but no additional anatomic abnormalities or fetal hydrops.

The mother underwent serial amnioreduction for severe polyhydramnios (amniotic fluid index >35 cm) at 27, 29, 31, and 33-weeks. At 33-weeks, she had preterm premature rupture of membranes (PPROM) and underwent emergent EXIT procedure. The fetal head and neck were delivered via a low-transverse hysterotomy. Otolaryngology performed a direct laryngoscopy and intubated the fetus without complications and the fetus was delivered uneventfully.

**Figure 2:**
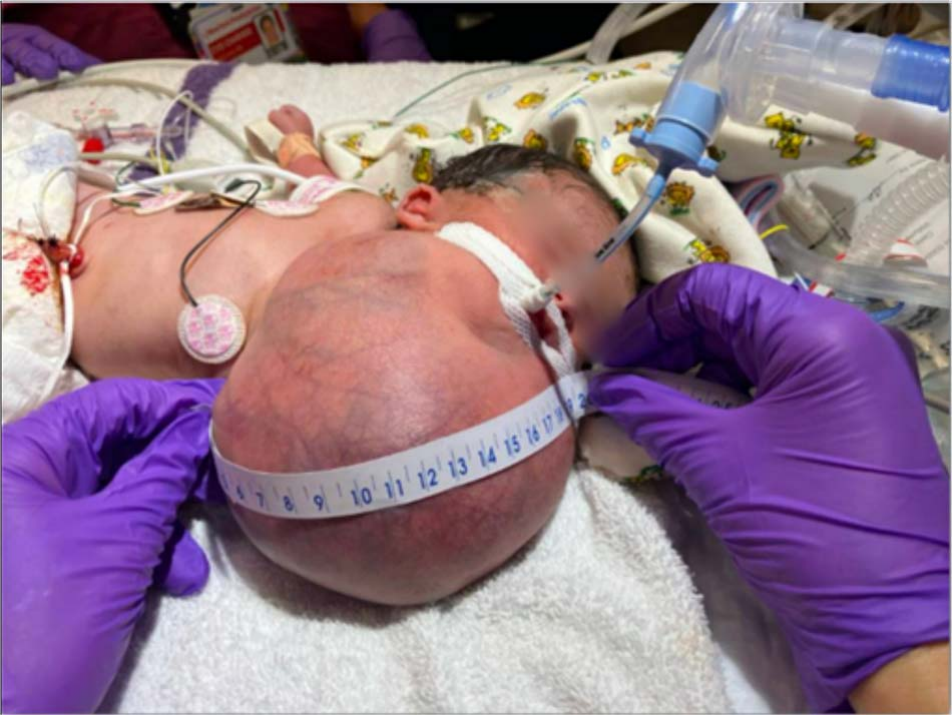
Image of newborn with large cervical mass measuring 17 × 19 cm.

**Figure 3:**
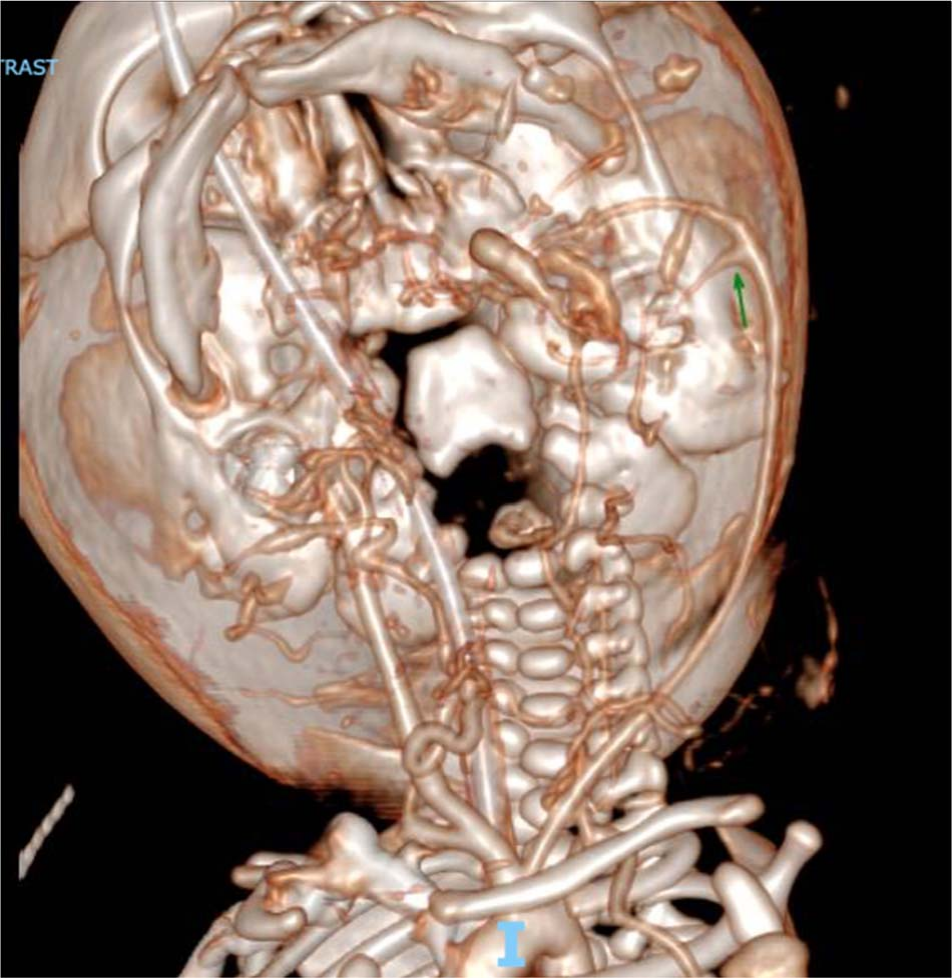
CT angiogram of the head with intravenous contrast demonstrating a hypervascular mass with arterial feeders originating from the left thyrocervical trunk, left external carotid artery, and right external carotid artery.

Physical examination of the 2.5 kg female revealed a 17 × 19 cm anterior cervical mass (Fig. 2). Labwork showed elevated alpha-fetoprotein and normal beta-human chorionic gonadotropin and inhibin levels. Head and neck imaging was consistent with a diagnosis of cervical teratoma. On CT angiography, the mass had a dominant arterial feeder from the left external carotid artery and additional arterial feeders from the left thyrocervical trunk and right external carotid artery (Fig. 3). The cystic components of the cervical teratoma continued to enlarge and required drainage at 3 and 5 days of life (DOL) totaling 350 ml. Cytology of the aspirate was non-diagnostic.

**Figure 4:**
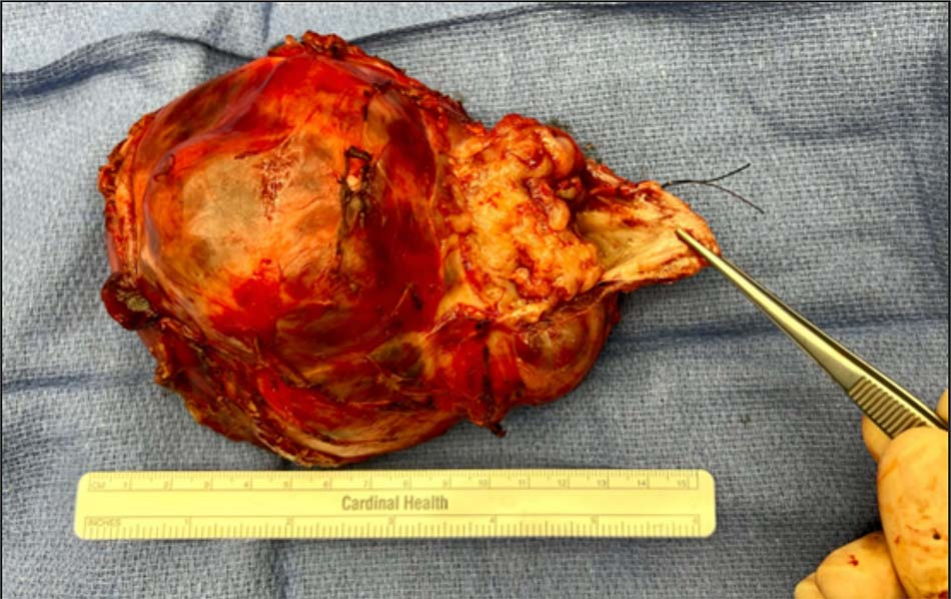
Image of resected teratoma.

At 8 DOL, the patient underwent teratoma resection (Fig. 4). The operative team included pediatric surgery, pediatric otolaryngology, adult head and neck surgical oncology, and plastic surgery. Intraoperative nerve monitoring of the cranial nerves VII, X-XII and the right recurrent laryngeal nerve was conducted. The lateral aspect of the mass was well encapsulated. It was dissected free from the overlying skin and surrounding critical structures including the carotid arteries, left vertebral artery, left recurrent laryngeal nerve, vagus nerves, esophagus, and trachea. The mass was densely adherent to the anterior pharynx with no capsular separation between the two structures. To obtain a gross total resection, a pharyngotomy was performed and repaired under visualization with direct laryngoscopy. During the procedure, four transient episodes of acute oxygen desaturation occurred. Three episodes resolved with positive pressure ventilation, steroids, albuterol, and manual stabilization of the endotracheal tube. One episode was accompanied by significant bradycardia which improved after 30 s of epinephrine and chest compressions. The patient was transferred to the neonatal ICU in stable condition after the procedure.

Final pathology demonstrated immature and mature teratoma. Pharyngeal repair was intact on direct laryngoscopy at one week postoperatively. Neonatal intensive care unit (NICU) course was notable for multiple failed extubations due to airway edema and granulation tissue, ultimately requiring tracheostomy. Post-surgical hypothyroidism was well-controlled with levothyroxine, and the patient was eventually discharged to a rehabilitation center with a tracheostomy collar and a gastrostomy tube. Continued apneic episodes prompted ENT evaluation and return to the operating room for supraglottic stenosis. At a 15-month follow-up, the infant demonstrated adequate weight gain via gastrostomy tube and was producing voice. Apart from left-sided vocal cord paralysis, no neurological deficits have been noted.

## Discussion

We report the successful management of one of the largest congenital cervical teratomas documented in the literature. The positive outcome for this patient is attributed to early prenatal detection with close surveillance and timely interventions, prompt mobilization of an experienced EXIT team, and meticulously choreographed multidisciplinary postnatal surgical management [9].

Barrette et al. conducted a review of 52 antenatally diagnosed cervical masses and found that the mean gestational age at diagnosis was 29 weeks 3 days [6]. This case demonstrates the benefits of earlier identification of fetal airway masses. The positive outcome for this patient may be attributed to early prenatal detection (20-weeks gestation), rigorous monitoring, and timely mitigation of poor prognostic factors beginning in the second trimester, when the mother underwent four amnioreduction procedures for severe polyhydramnios providing the foetus critical weeks of development in utero. Due to foetal characteristics significantly associated with requiring an emergent airway at birth, an EXIT procedure was planned and successfully executed at 33-weeks gestation, even in the setting of PPROM requiring an emergent delivery [6, 7].

**Figure 5:**
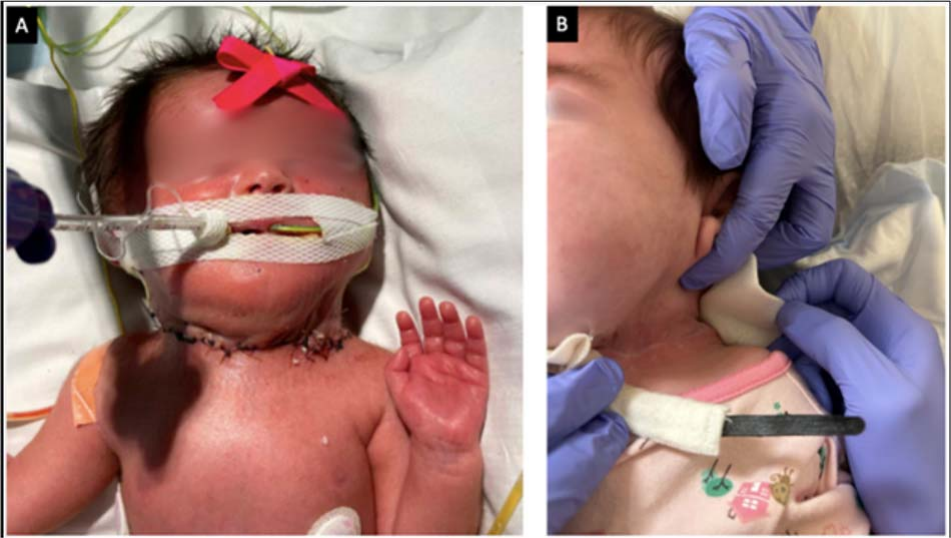
(A) Image of the cervical incision on post-op Day 6. (B) Image of patient’s incision at a 5-month follow-up visit.

This case highlights the importance of a multidisciplinary approach in managing foetal airway masses [7, 10, 11]. This congenital cervical teratoma was 12-fold larger than the median reported volume of congenital head and neck teratomas (72 ml) [12]. The size and complexity of this cervical teratoma with involvement of the pharynx and significant laryngeal compression required the expertise of radiology, pediatric surgery, pediatric otolaryngology, and adult head and neck surgical oncology to resect safely [13]. Intraoperative nerve monitoring was crucial for safe resection. Plastic surgery provided functional and aesthetic reconstruction of the distorted anatomical structures after the teratoma was resected (Fig. 5).

The goal of this report is to help guide the prenatal and postnatal management of patients with complex head and neck masses to ensure future successful outcomes.
